# A novel multiplex PCR for detection of *Pseudomonas aeruginosa*: A major cause of wound infections

**DOI:** 10.12669/pjms.294.3652

**Published:** 2013

**Authors:** Muhammad Salman, Aamir Ali, Abdul Haque

**Affiliations:** 1 Muhammad Salman BS, Health Biotechnology Division, National Institute for Biotechnology and Genetic Engineering, Faisalabad affiliated with Pakistan Institute of Engineering and Applied Sciences (PIEAS), Islamabad, Pakistan.; 2 Dr. Aamir Ali, PhD, Health Biotechnology Division, National Institute for Biotechnology and Genetic Engineering, Faisalabad affiliated with Pakistan Institute of Engineering and Applied Sciences (PIEAS), Islamabad, Pakistan.; 3 Dr. Abdul Haque, PhD, Health Biotechnology Division, National Institute for Biotechnology and Genetic Engineering, Faisalabad affiliated with Pakistan Institute of Engineering and Applied Sciences (PIEAS), Islamabad, Pakistan.

**Keywords:** Multiplex PCR, wound infections, *P. aeruginosa*

## Abstract

***Background and Objective: ***Wound infections are often difficult to treat due to various bacterial pathogens. *Pseudomonas aeruginosa* is one of the common invaders of open wounds. Precise diagnosis of this etiological agent in wound infections is of critical importance particularly in treatment of problematic cases. The existing diagnostic methods have certain limitations particularly related to specificity. Our objective was to to establish a comprehensive and reliable multiplex PCR to confirm diagnosis of *P. aeruginosa*.

***Methods: ***A multiplex PCR test was developed for rapid and comprehensive identification of *P. aeruginosa*. Four highly specific genes were targeted simultaneously for detection of genus, species and exotoxin production (*16S rDNA, gyrB, oprL *and* ETA*) in *P. aeruginosa*; additionally one internal control gene (*invA*) of *Salmonella *was used. The specificity of the multiplex PCR was confirmed using internal and negative controls. Amplified fragments were confirmed by restriction analysis and DNA sequencing.

***Results: ***The developed method was applied on 40 morphologically suspected *P. aeruginosa* isolates (from 200 pus samples) and 18 isolates were confirmed as* P. aeruginosa*. In comparison, only 12 could be identified biochemically.

***Conclusions: ***Combination of the four reported genes in multiplex PCR provided more confident and comprehensive detection of *P. aeruginosa* which is applicable for screening of wound infections and assisting treatment strategy.

## INTRODUCTION

Wound infections are complications caused by bacteria which result in belated healing and can sometimes be even life-threatening.^1^ These infections also considerably contribute to increased health care costs.^2^ *Pseudomonas aeruginosa* (*P. aeruginosa*) has been recognized as a frequent inhabitant of chronic non-healing wounds^3^ and is one of the foremost opportunistic bacteria isolated from wounds which cause high morbidity and mortality despite antimicrobial therapy.^4^ *P. aeruginosa *infections are generally detected by standard microbiological techniques such as phenotypic and biochemical profiles,^5^ however these commercial tests tend to be lengthy **and **unreliable.^5^^,^^6^ Molecular techniques, such as polymerase chain reaction (PCR) are rapid and reliable for the identification of microbial pathogens,^6^ many PCR based diagnostic methods have been developed for *P. aeruginosa*.^7^^-^^9^

However, most of the protocols target only a single gene fragment which is inadequate for comprehensive and reliable diagnosis.^9^^,^^10^ The reason is that *P. aeruginosa* strains from patients demonstrate high genotypic diversity^11^ and several studies have confirmed the absence of one or more of the virulence genes in some *P. aeruginosa* strains.^12^ To overcome these problems, several multiplex PCR protocols have been reported,^13^ but due to genetic exchanges among *P. aeruginosa* and closely related bacteria, most multiplex PCRs have low specificity.^14^ Therefore, there is need to establish a comprehensive and reliable multiplex PCR to confirm diagnosis of *P. aeruginosa*.

This study deals with optimization of a multiplex PCR targeting four different gene fragments specific for *P. aeruginosa* (*16S rDNA, gyrB, oprL *and* ETA*) simultaneously for comprehensive and confirmatory identification. Application of this method on clinical samples demonstrated improved efficiency and reproducibility.

## METHODS

***Bacterial isolation:*** *P. aeruginosa* strain # MS6 (previously confirmed as *P. aeruginosa*) was taken from National Institute for Biotechnology and Genetic Engineering (NIBGE) stock cultures. Two hundred wound (pus) samples were collected from non-hospitalized outdoor patients from Allied Hospital, Faisalabad, Pakistan in the year 2011. Samples were collected on sterile cotton wool swabs which were transported to laboratory immediately. In case of a delay, the samples were kept at 4°C in tryptic soy broth (TSB) till transportation. *Salmonella enterica* serovars Typhi (as internal control) and isolates of* Staphylococcus aureus, Escherichia coli, Klebsiella aerogenes, Proteus vulgaris* and *Proteus mirabilis* were also taken from NIBGE stock cultures and used as negative controls. The swabs were streaked on MacConkey agar plates and kept overnight at 37°C to observe colony morphology. Five different colonies from each of the MacConkey agar plates, suspected as *P. aeruginosa*, were processed further for biochemical identification using RapidONE Remel kit (Thermo Fisher Scientific, Kansas, USA) according to manufacturer’s instructions.

***DNA extraction: ***Morphologically identified *P. aeruginosa* from wound samples, *P. aeruginosa* MS6, internal control (*S*. Typhi) and negative controls strains were cultured in TSB. Genomic DNA of the overnight cultures was extracted by the conventional phenol-chloroform method.^15 ^Integrity of the DNA samples was checked by electrophoresis on 1% agarose gel and purity was determined by ratio of A260/A280 using a spectrophotometer (Spectro22 Labomed 22. Inc, USA).

***PCR amplification: ***First set of primers (Pa16S-F and Pa16S-R) was specific for *16s rDNA* gene of the genus *Pseudomonas*.^14^ Second (*gyrB*-F and *gyrB*-R) and third (*oprL*-F and *oprL*-R) primer sets targeted species gene sequences of *gyrB* and *oprL* genes respectively.^9^^,^^7^ Fourth set (ETA-F and ETA-R) was used for amplification of exotoxin production related gene fragment (*ETA*).^16^ For internal control, the *invA* gene of *S*. Typhi was targeted using *invA*-F and *invA*-R primers.^17^ All sets of oligonucleotide primers were synthesized by Gene link (New York, USA). Sequences and lengths of targeted gene fragments are given in Table-I. Preliminarily, the PCR amplification conditions were optimized with *P. aeruginosa *MS6 for each of the four genes separately and then for the multiplex PCR.

Each 50μl of the multiplex PCR mixture, in addition to the template DNA, contained 10 x buffer 5 μl, 1.5 mM MgCl_2_, 0.4mM of each dNTP, 5U of *Taq* DNA polymerase (Fermentas, USA), 0.25 μM of the primers targeting *oprL* gene and 0.5μM of each of the primers targeting *invA, gyrB, ETA *and* Pa16S* gene fragments. The thermal cycler (PTC 06 ICCC, Pakistan) conditions for the multiplex PCR were: 94°C for 5 minutes followed by 35 cycles of 94°C for 1 min, 58°C for 1 min, 72°C for 1.5 minutes; and a final extension step at 72°C for 7 minutes. Similar multiplex PCR conditions were applied to the DNA templates of negative control isolates. On completion of PCR cycles, the amplified products were electrophoresed on 2% agarose gel, stained with ethidium bromide (5 μg /100 ml) and visualized under UV illumination and documentation system (Viopro platinum, Uvitech, Cambridge, UK). The optimized multiplex PCR conditions were applied on morphologically identified clinical wound samples.

***Restriction analysis: ***The amplified products of the targeted genes, *gyrB*, *ETA*, o*prL* and *16S rDNA*, were subjected to restriction analysis with site specific restriction endonucleases. *Bsu*RI was used to restrict amplified products of *gyrB* and *16S rDNA* while *Cf*rI and *Nco*I restriction endonucleases were used for *ETA* and *oprL* respectively. Each of the restriction mixtures contained 5µl (10 U) of enzyme, 3 µl of enzyme buffer, 8 µl of PCR-amplified product and 18µl of deionized water followed by overnight incubation at 37°C. Restricted fragments were electrophoresed on 2.5% agarose gel and visualized under UV illumination and documentation system (Viopro platinum, Uvitech, Cambridge, UK).

***DNA sequencing: ***Sequencing of the amplified gene fragments of *P. aeruginosa* MS6 (*gyrB, ETA, oprL* and *16S rDNA*) was done by a commercial vendor, Macrogen Inc. (Seoul, Korea) and the sequencing chromatographs were analyzed with Seqman software (DNASTAR, Inc. Wisconsin, USA) followed by submission to GenBank with accession numbers [GenBank: JN717229, GenBank: JN717230, GenBank: JN717231, GenBank: JN717232] respectively.

## RESULTS

**Fig.1:**
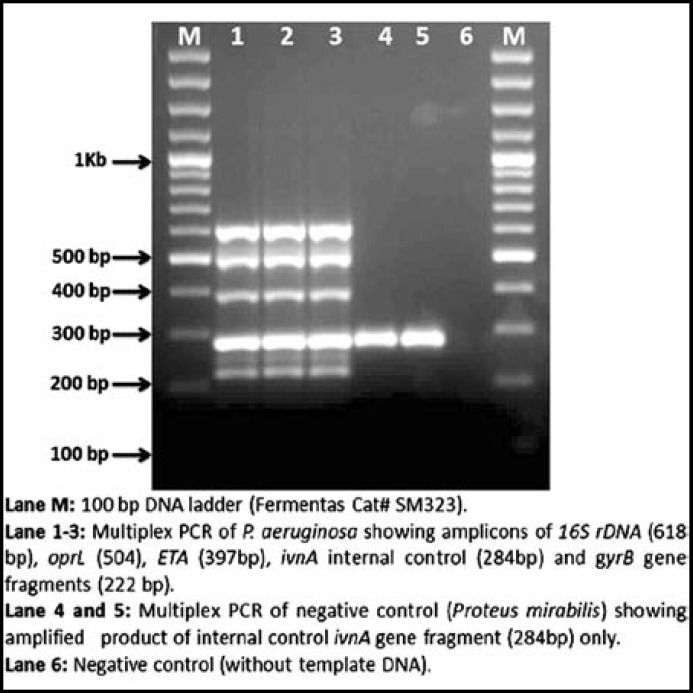
Multiplex PCR of *P. aeruginosa* isolates. Lane M: 100 bp DNA ladder (Fermentas Cat# SM323). Lane 1-3: Multiplex PCR of *P. aeruginosa* showing amplicons of 16S rDNA (618 bp), oprL (504), ETA (397bp), *ivnA* internal control (284bp) and gyrB gene fragments (222 bp). Lane 4 and 5: Multiplex PCR of negative control (*Proteus mirabilis*) showing amplified product of internal control *ivnA* gene fragment (284bp) only. Lane 6: Negative control (without template DNA).

*Identification of P. aeruginosa:* **From 200 clinical pus samples, 40 isolates were suspected as** ***P. aeruginosa*** **on the basis of formation of large, translucent, pale, mucoid colonies on MacConkey agar plates. On nutrient agar, typical greenish blue color (due to production of pyocanin and fluorescin pigments) was observed spreading throughout the medium. Biochemically, 12 isolates were identified as** ***P. aeruginosa*** **by RapidONE Remel kit (Thermo Fisher Scientific, Kansas, USA). However, the number of positive isolates increased from 12 to 18 by multiplex PCR developed for this study. In all cases, amplification products of internal control (284 bp), specific *P. aeruginosa* gene fragments *gyrB* (222 bp)*, ETA *(397 bp)*, oprL *(504 bp) and* 16S rDNA* (618 bp) were obtained (**Fig.1**). There was no amplification in case of negative control bacteria.**

**Fig.2:**
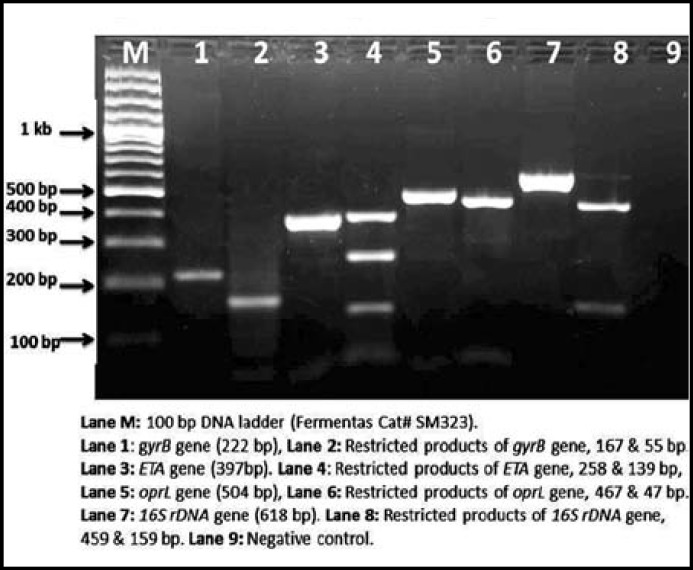
Restriction analysis of *P. aeruginosa* MS6 strain. Lane M: 100 bp DNA ladder (Fermentas Cat# SM323). Lane 1: *gyrB* gene (222 bp), Lane 2: Restricted products of *gyrB* gene, 167 & 55 bp. Lane 3:ETA gene (397bp). Lane 4: Restricted products of *ETA* gene, 258 & 139 bp. Lane 5: *oprL* gene (504 bp), Lane 6: Restricted products of *oprL* gene, 467 & 47 bp. Lane 7:16S *rDNA* gene (618 bp). Lane 8: Restricted products of 16S *rDNA *gene, 459 & 159 bp. Lane 9: Negative control

***Restriction analysis and DNA sequencing: ***Restriction of all the four amplified gene fragments resulted in their cleavage into smaller fragments confirming band sizes as retrieved from website www.nebcutter.com. The *16S rDNA *gene product (618 bp) produced 459 and 159 bp fragments, the *oprL* gene product (504 bp) was cleaved into 467 and 47 bp fragments, the *ETA* gene product (397 bp) yielded 258 and 139 bp fragments and the *gyrB* gene product (222 bp) was cleaved into fragments of 167 and 55 bp (Fig.2). The sequences of the amplified gene fragments (*gyrB, ETA, oprL* and *16S rDNA*) of *P. aeruginosa* strain # MS6 were compared with already reported gene sequences of *P. aeruginosa *on NCBI database using BLAST search and top two similarity results were noted. The *gyrB* gene fragment sequence was found 99% identical with PAO1 [GenBank: AE004091.2] and 98% identical with NCGM2 [GenBank: AP012280.1]. The *ETA *gene was found 99% identical with PAO1 [GenBank: AE004091.2] and 98% identical with NCGM2 [GenBank: AP012280.1]. The o*prL* gene fragment sequence was found 99% identical with each of PAO1 [GenBank: AE004091.2] and M18 [GenBank: CP002496.1]. The *16S rDNA* gene fragment sequence was found 97% identical with each of ZDC-2 [GenBank: JQ249910.1] and CW512 [GenBank: FM207514.1].

## DISCUSSION

Wound infections often become complicated and problematic due to invasion of multiple organisms and most of them are multi drug resistant. One of the major causes of complications in the wound infections is *P. aeruginosa* and its early and precise diagnosis is of substantial importance.^8^ The delay in accurate diagnosis may prolong the hospitalization and effective treatment.^5^ In routine, the microbiological culture is the mainstay for detection of *P. aeruginosa*. Although some other detection methods promise better sensitivity but these methods still need evaluation and validation,^14^^,^^16^ because *Pseudomonas* species are sometime indistinguishable from other closely related microbes.^18^ The biochemical tests lack specificity as in one study, 52 non-typical *P. aeruginosa* isolates were not identified by API 20 E kit.^19^ We had similar observations with Rapid ONE Remel kit during this study.

**Table-I:** Primers used in multiplex PCR

*Primers*	*Sequences (5* ^/^ * - 3* ^/^ *)*	*Genes*	*Amplicon size (bp)*
gyrB-F	CCTGACCATCCGTCGCCACAAC	*gyrB*	222^9^
gyrB-R	CGCAGCAGGATGCCGACGCC		
ETA-F	GACAACGCCCTCAGCATCACCA	*ETA*	397^16^
ETA-R	CGCTGGCCCATTCGCTCCAGCG		
oprL-F	ATG GAAATGCTGAAATTCGGC	*oprL*	504^7^
oprL-R	CTTCTTCAGCTCGACGCGACG		
Pa16S-F	GGGGGATCTTCGGACCTCA	*16SrDNA*	618^14^
Pa16S-R	TCCTTAGAGTGCCCACCCG		
invA-F	GTGAAATTATCGCCACGTTCGGGCAA	*invA*	284^17^
InvA-R	TCATCGCACCGTCAAAGGAACC		

Many researchers have made attempts to develop molecular methods especially PCR for the detection of *P. aeruginosa,*^20^ but due to various limitations such as genetic diversity and the fact that genome sequences of closest species are not available, a comprehensive and definitive methodology is still lacking.

A multiplex PCR on respiratory samples targeting 3 genes (*algD, chit A* and *16S rDNA*) detected *P. aeruginosa* in 78.7% samples whereas culture was positive in 56% cases only.^21^ A real time PCR on multiple targets for identification of *P. aeruginosa* reported the lesser specificity of *ETA* and *algD* genes as compared to *oprl*.^5^ Similar results about lesser specificity of *ETA* and *algD* genes have also been reported by other researchers.^21^ A study using quantitative PCR (qPCR) to target the o*prL* gene showed 85% specificity and concluded that qPCR may have a predictive value for impending *P. aeruginosa* infection for only a limited number of patients.^22^ The specificity of another developed multiplex PCR was reported as only 45.5%.^23^ *P. aeruginosa* from various sources were studied and it was found that the combination of *oprl, oprL, 16S rDNA*, *ETA* and *fliC* genes leads to false positive result because of genetic conservation and it showed difficulties in building up a reliable screening with these targets, however the *gyrB* and *16S-23S rDNA ITS* genes were highly specific.^7^

We selected four highly specific gene fragments of *P. aeruginosa* (*16S rDNA, gyrB, oprL *and* ETA*) and optimized a multiplex PCR for its comprehensive and reliable identification with 100% specificity as no amplification was found in case of negative controls. The specificity of each of the four targeted genes has been reported earlier in different studies separately. The *16S rDNA* gene showed 96.5% specificity using real time PCR.^24^ The *gyrB *gene coding a type II topoisomerase has also been noted to be a better candidate for the identification of bacterial species.^   25^ The specificity of the *oprL* gene based PCRs for *P. aeruginosa* has also been reported as 80%.^7^

After multiplex PCR, amplicons were confirmed by restriction analysis which provided the relevant fragments of exact sizes and the nucleotide sequencing of the four targeted gene fragments showed more than 97% identity with *P. aeruginosa* strain PAO1. Main benefit of this developed multiplex PCR is the detection of *gyrB, ETA, OprL*, and *16S rDNA* genes simultaneously that, in presence of internal control, eliminates the chances of false positive results which are due to phenotypic and monogenic resemblance of *P. aeruginosa* with closely related species.

## CONCLUSIONS

We conclude that the unique combination of these four genes in multiplex PCR provides more confident and reliable detection of *P. aeruginosa* for screening of wound infections that can be helpful to the clinicians for effective antimicrobial therapy.

## Authors contribution

*MS:* Main lab work and preparing draft of paper. *AA:* Practical guidance in molecular and microbiology work. *AH:* Concept and finalization of manuscript.
